# Prognostic Significance and Clinicopathological Associations of COX-2 SNP in Patients with Nonsmall Cell Lung Cancer

**DOI:** 10.1155/2009/139590

**Published:** 2009-11-22

**Authors:** Peter P. Grimminger, Jan Stöhlmacher, Daniel Vallböhmer, Paul M. Schneider, Arnulf H. Hölscher, Ralf Metzger, Peter V. Danenberg, Jan Brabender

**Affiliations:** ^1^Department of General, Visceral and Tumor Surgery, University of Cologne, 50931 Cologne, Germany; ^2^Internal Medicine Clinic I, Carl Gustav Carus University Hospital, Dresden, Germany; ^3^Department of Visceral and Transplant Surgery, University Clinic Zürich, Zurich, Switzerland; ^4^Faculty of Biochemistry and Molecular Biology, University of Southern California, Los Angeles, CA 90089, USA

## Abstract

*Background*. To further improve the screening, diagnosis, and therapy of patients with nonsmall cell lung cancer (NSCLC) additional diagnostic tools are urgently needed. Gene expression of Cyclooxygenase-2 (COX-2) has been linked to prognosis in patients with NSCLC. The role of the COX-2 926G>C Single Nucleotide Polymorphism (SNP) in patients with NSCLC remains unclear. The aim of this study was to investigate the potential of the COX-2 926G>C SNP as a molecular marker in this disease. *Methods*. COX-2 926G>C SNP was analyzed in surgically resected tumor tissue of 85 patients with NSCLC using a PCR-based RFLP technique. *Results*. The COX-2 926G>C SNP genotypes were detected with the following frequencies: GG *n* = 62 (73%), GC *n* = 20 (23%), CC *n* = 3 (4%). There were no associations between COX-2 SNP genotype and histology, grading or gender detectable. COX-2 SNP was significantly associated with tumor stage (*P* = .032) and lymph node status (*P* = .016, Chi-square test). With a median followup of 85.9 months, the median survival was 59.7 months. There were no associations seen between the COX-2 SNP genotype and patients prognosis. *Conclusions*. The COX-2 926G>C SNP is detectable at a high frequency in patients with NSCLC. The COX-2 926G>C SNP genotype is not a prognostic molecular marker in this disease. However, patients with the GC or CC genotype seem more susceptible to lymph node metastases and higher tumor stage than patients with the GG genotype. The results suggest COX-2 926G>C SNP as a molecular marker for lymph node involvement in this disease.

## 1. Introduction

The smoking of tobacco is the most prevalent cause of lung cancer, which is the leading cause of cancer mortality in the world towards the end of the 20th century [1, 2]. Each year approximately 200 000 new cases of lung cancer are diagnosed in the United States, and there were over 160 000 lung cancer related deaths in 2008 [3]. The only treatment to cure patients with nonsmall cell lung cancer (NSCLC) is radical surgery, but the 5-year survival rate still remains poor. However, the concept of individualized treatment based on genetic differences among patients promises to provide improved treatment outcomes. Thus, the molecular mechanisms involved in carcinogenesis and pharmacogenetics have to be studied so that tailored treatments can be discovered and developed.

Inflammation has been recognized as a contributing factor in pathogenesis of many cancers [4]. Epidemiologic studies have shown that prolonged use of nonsteroidal anti-inflammatory drugs (NSAIDs) reduces the risk of a variety of cancers including lung cancer [5–8]. Cyclooxygenases (COXs, also named prostaglandin endoperoxide synthases or PTGSs) are the key enzymes in the conversion of arachidonic acid to prostaglandin (PG) and other eicosanoids [9, 10]. Two isoforms have been identified; COX-1 is consistently expressed in nearly all cells whereas COX-2 is normally undetectable but induced under circumstances such as inflammation and cancer [11]. Overexpression of COX-2 has been reported in several cancers, such as colorectal [12, 13], pancreatic [14], breast [15], esophageal [16], gastric [17], lung [18, 19], and several other cancers [20–23].

Single nucleotide polymorphisms (SNPs) are common in the human genetic pool, and there is growing evidence suggesting that genetic polymorphisms play a role in the variability of drug response and toxicity in patients. A predictive value concerning the response to chemotherapy treatment has been reported for certain genetic SNPs in several tumors, for example, gastric cancer [24], breast cancer, colorectal cancer [25–27], and lung cancer [28, 29]. Earlier studies have already shown that COX-2 SNPs have an impact in promoter activity and therefore influence the variability of response [30]. Papafili et al. reported a transcription alteration of the COX-2 gene caused by the COX-2 926G>C SNP in the promoter region and an increase of the levels of C-reactive protein.

The COX-2 926G>C SNP has been investigated earlier regarding a possible increased risk of developing NSCLC, but no association could be found for this SNP [31]. No study of COX-2 926G>C polymorphism and potential prognostic significance in NSCLC cancer patients has been reported so far. Hence the rationale for conducting this study was to investigate a possible prognostic role of the COX 926G>C SNP in NSCLC.

## 2. Material and Methods

### 2.1. Patients

85 tumor specimens from NSCLC patients, available from a previous prospective clinical trail of 103 consecutive patients [32], were included in this study. 65 patients were male (76%) and 20 female (24%) with the median age of 62.4 years. Seventy-six (89%) of these patients were smokers.

According to the International Union Against Cancer (UICC) TNM classification [33], 42 patients (49%) were tumor stage I, 18 patients (21%) stage II, and 25 patients (30%) stage IIIA. 39 (46%) patients had squamous cell carcinoma, 31 (36%) had adenocarcinoma, and 15 (18%) had large cell carcinoma. All 85 patients underwent thoracic surgery, and the tumors were R0 resected. Patients with histopathologic stage 3a tumors received postoperative radiotherapy. Informed consent was obtained from each patient.

The median followup was 85.9 months (range 63–105), and no patient was lost to followup. Tissue for gene expression analysis was obtained during surgery immediately after lung resection and before starting mediastinal lymphadenectomy. The tissues were immediately frozen in liquid nitrogen and stored at −80°C. Six-micrometer frozen sections were taken from blocks of tumor tissue. Starting with the first section, every fifth section was routinely stained with hematoxylin and eosin and evaluated histopathologically. Sections were pooled for analysis from areas estimated to have at least 75% malignant cells.

The primary tumors were graded histopathologically as well differentiated (G1, one patient), moderately differentiated (G2, eighteen patients), and poorly differentiated (G3, sixty-six patients).

### 2.2. DNA Extraction and Genotyping of the COX-2 926G>C SNP

DNA was extracted from representative tumor sections using the QIAamp DNA Mini Kit (Qiagen, Hilden, Germany). The COX-2 926G>C polymorphism was analysed in tumor tissue of 85 patients with NSCLC using a PCR-based RFLP technique. Forward and reverse primers used were as follows: 5′-CAT TTA GCG TCC CTG CAA AT-3′ and 5′-TAC CTT CAC CCC CTC CTT GT-3′. Briefly, an approximately 2 ng DNA was added to a reaction volume of 15 *μ*L, containing 7.5 *μ*L TaqMan Universal PCR Master Mix, No AmpErase UNG, and 0.75 *μ*L custom-designed probe. Amplifications and determination of genotypes were performed using an Applied Biosystems 7500 Real Time PCR System as follows: 95°C (10′), 45 cycles of 93°C (15′′), and 60°C (1′). PCR fragments were digested using 3 units of the restriction enzyme AciI and separated on a 3% agarose gel. A technician blinded for the clinical data performed PCR/RFLP analyses. A random sample of 20% of each polymorphism was repeated and showed 100% concordance.

### 2.3. Statistical Analysis

Statistical analyses were carried out using SPSS for Windows (version 17.0). A *χ* ^2^ test was used to assess the association between categorical clinicopathologic data and COX-2 926G>C SNP genotype. Hazard ratios were used to calculate the relative risks of death. These calculations were based on the Pike estimate, with the use of the observed and expected number of events as calculated in the log-rank test statistic. The log-rank test [34] and Kaplan-Meier plots [35] were used to evaluate the association of genotypes and overall survival. Multivariate analysis was performed with the Cox proportional hazards regression model. Statistical significance was interpreted as *P* < .05. All *P*-values reported were based on two-sided tests.

## 3. Results

In the studied cohort, the COX-2 926G>C SNP genotypes were detected with the following disposition: the wild type (WT) GG in *n* = 62 (73%), the heterozygote SNP GC in *n* = 20 (23%), and the homozygote SNP CC in *n* = 3 (4%).

No association between COX-2 926G>C SNP genotype and histology was seen, even though the CC genotype (*n* = 3) was only found in squamous cell carcinoma patients.

**Figure 1:**
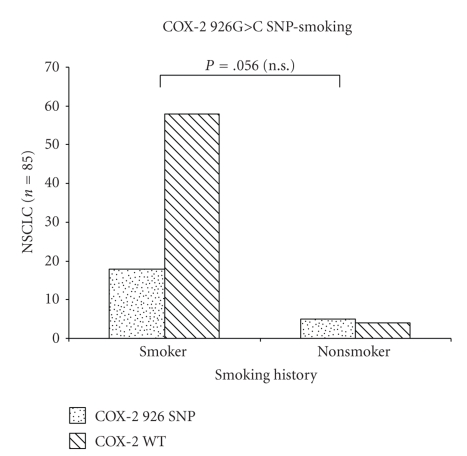
Distribution of the COX-2 926G>C SNP and wild type in smoker or nonsmoker. In nonsmoker a COX-2 926 polymorphism is borderline significant and more frequent than in smokers (*P* = .056 Fisher's Exact Test).

Also, neither grading nor gender had any detectable association with the COX-2 926G>C SNP. All three patients with the genotype CC were male, but due to the small number of the CC genotype there was no statistical significance. Seventy six (89%) of the patients were smokers. There was no association between smokers and different COX 926 polymorphisms. However, in nonsmokers, the COX 926 polymorphism is more frequent than in smokers with borderline significance (*P* = .42 Pearson *χ* ^2^; *P* = .056 Fisher's Exact Test) (Figure 1).

**Figure 2:**
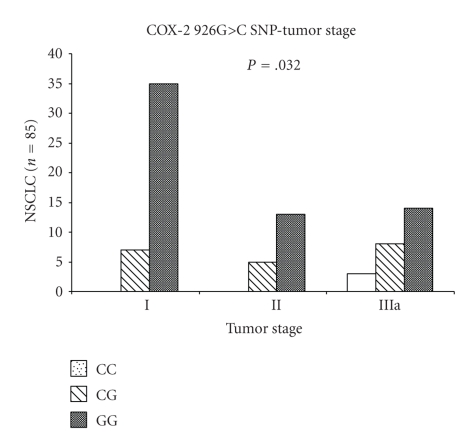
Tumor stage and COX-2 926G>C SNP in NSCLC. The COX-2 926G>C SNP was significantly associated with a higher tumor stage (*P* = .032, Pearson *χ* ^2^ test).

The COX-2 926G>C SNP was significantly associated with a higher tumor stage (*P* = .032, Pearson *χ* ^2^ test) (Figure 2). All three CC genotypes were stage IIIa, 5 CG and 13 GG genotypes were stage II, and 7 CG and 35 GG genotypes were found in stage I.

**Figure 3:**
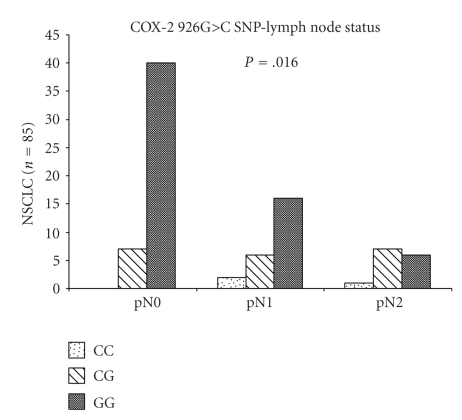
Distribution of the COX-2 926G>C polymorphism and lymph node status. The COX-2 926G>C polymorphism is associated with a higher lymph node status (*P* = .016, *χ* ^2^ test).

Also, associations were discovered in the COX-2 926G>C polymorphism and the lymph node metastasis (*P* = .016, *χ* ^2^ test) (Figure 3). The three patients with the CC genotype had all lymph node metastasis, two were pN1 and one was pN2. Fifteen out of 20 patients (75%) with the CG genotype had lymph node metastasis. Six of these patients were pN1 and seven pN2. Only 35% of the patients (22 of 62) with the GG genotype were suffering from lymph node metastasis.

**Figure 4:**
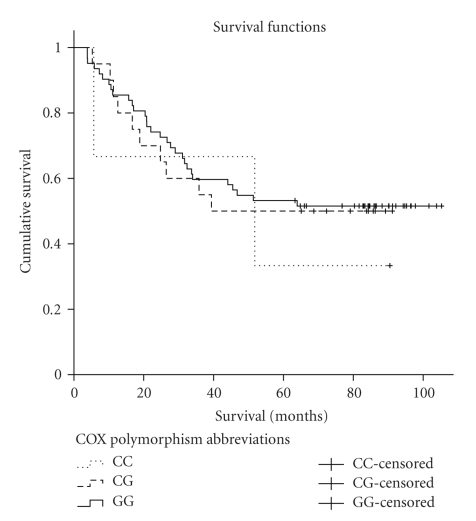
Kaplan-Meier cumulative survival plot for nonsmall cell lung cancer patients with CC, CG, and GG COX-2 926G>C SNP. The probability of survival was not statistically different between the different genotypes.

With a median followup of 85.9 months, the median survival was 59.7 months (range 38–105 months). Neither the log-rank test (Mantel-Cox) (*P* = .848) nor the Kaplan-Meier plots (Figure 4) showed any prognostic significance for the COX-2 926G>C SNP. 

## 4. Discussion

The COX-2 pathway is important in cancer development because it is involved in the regulation of various critical cellular processes such as tumor progression, metastases, angiogenesis, and chemotherapy resistance [36–39]. Elevated COX-2 expression has been associated with poor prognoses in lung [40–42] and other cancers, such breast [43], head and neck [44], colon [45], and cervix carcinomas [46]. However, little is known about COX-2 single nucleotide polymorphisms in NSCLC. In this study, we found that the COX-2 926G>C SNP is detectable at a high frequency in patients with NSCLC. We used PCR-based RFLP protocols to analyze the COX-2 926 genotype and found that 73% of the patients had the wild type genotype (GG), 23% were heterozygote (GC) and 4% homozygote (CC) for the COX-2 926 polymorphism.

Several previous studies have examined the associations of COX-2 polymorphisms and tumor diseases. In breast cancer, the COX-2 169-GG genotype was associated with increased risk [47], but the COX-2 926G>C SNP was not [48], while some tenuous evidence was found for an interaction between the C allele of the COX-2 8473 SNP with NSAIDs to reduce risk for hormone receptor positive breast cancer. In the case of NSCLC, it was suggested that the COX-2 8473SNP is associated with an increased risk of developing lung cancer [31] but COX-2 926G>C SNP was not, as already mentioned in the introduction.

The association between smoking and the COX-2 926G>C SNP did not reach statistical significance in this study but the trend suggests the need for further investigation with larger numbers.

Although we did not find the COX-2 926G>C SNP to be a prognostic marker for NSCLC, NSCLC patients with the GC or CC genotype were apparently more susceptible to lymph node metastases and higher tumor stage than patients with the GG genotype, suggesting that the COX-2 926G>C SNP is a molecular marker for lymph node involvement.
